# Pharmacogenetic analysis of the effects of polymorphisms in *APOE*, *IDE *and *IL1B *on a ketone body based therapeutic on cognition in mild to moderate Alzheimer's disease; a randomized, double-blind, placebo-controlled study

**DOI:** 10.1186/1471-2350-12-137

**Published:** 2011-10-12

**Authors:** Samuel T Henderson, Judes Poirier

**Affiliations:** 1 Accera Inc., 380 Interlocken Crescent, Ste 780, Broomfield, CO, 80021, USA; 2 Douglas Institute Research Centre, 6825 Lasalle, Verdun, H4H 1R3, Canada

**Keywords:** Alzheimer's disease, ketone bodies, *APOE*, *IDE*, *IL1B*, insulin, memory, cognition

## Abstract

**Background:**

To examine the effect of genetic variation in *APOE*, *IDE *and *IL1B *on the response to induced ketosis in the Alzheimer's Disease Assessment Scale-Cognitive subscale (ADAS-Cog) in subjects with mild to moderate Alzheimer's disease (AD).

**Methods:**

Genotype effects on ADAS-Cog scores from a randomized, double-blind, placebo-controlled study in mild to moderate AD were examined by an overall two way analysis of variance. In addition, interactions with the carriage status of the epsilon 4 allele of the *APOE *gene (*APOE4*) were examined.

**Results:**

Significant differences in response to induced ketosis were found among non-carriers of putative gain-of-function polymorphisms in rs1143627 and rs16944 in the *IL1B *gene and among variants of the polymorphism rs2251101 in the *IDE *gene. Significant differences were found among non-carriers of the *APOE4 *gene, with notable improvement among the E3/E3 genotype group.

**Conclusions:**

Variants in *APOE*, *IL1B *and *IDE *may influence the cognitive response to induced ketosis in patients with mild to moderate AD.

**Trial registration:**

This trial was registered with ClinicalTrials.gov, registry number NCT00142805.

## Background

Alzheimer's disease (AD) is a progressive neurodegenerative disease. The major risk factors for the most common form of AD, known as late onset or sporadic AD, are age and possession of one or more copies of the epsilon 4 variant of the apolipoprotein E gene (*APOE4*). *APOE4 *behaves in a dominant dose-dependent manner. One copy of *APOE4 *increases the risk of developing AD by about 3 fold, while two copies increases the risk approximately 10 fold [1,2].

Alzheimer's disease is characterized by an early and progressive decrease in the cerebral metabolic rate of glucose (CMRglc) [3-5]. The primary regions affected in AD are the posterior cingulate and the parietal, temporal, and prefrontal cortices. These regions correlate with the highly metabolically active default network, suggesting a metabolic link between hypometabolism, amyloid deposition, and cell atrophy (for review see [6]). The declines in CMRglc in AD could be attributed to loss of cells or synaptic fields, however, decreased rates of glucose phosphorylation [7] and low expression of energy generating genes [8] have been observed in AD, suggesting an underlying metabolic defect in these brain regions [9].

Under normal conditions, the brain is dependent almost exclusively on glucose and few other substrates are metabolized [10]. Therefore, declines in glucose utilization can result in severe impairment. Under conditions of low glucose availability, such as during fasting or low carbohydrate intake, the body will mobilize ketone bodies, from energy-rich fat stores, which can provide an alternative substrate for glucose metabolism in the brain [11]. The endogenously produced compounds β-hydroxybutyrate, acetoacetate and acetone are normally referred to as ketone bodies. Due to their efficient metabolism and ability to substitute for glucose, ketone bodies offer a potential therapeutic benefit for AD [12], as well as other neurological disorders [13].

Previous studies have demonstrated that the induction of ketosis in mild to moderate AD patients improves scores in the Alzheimer's Disease Assessment Scale -Cognitive subscale (ADAS-Cog) relative to placebo among non-carriers of the *APOE4 *allele using both acute [14] and chronic [15] dosing regimens. Despite the replication of these findings, it is unclear why E4(-) subjects would respond to ketosis while E4(+) subjects would not. Some evidence suggests that E4(+) subjects may have greater mitochondrial dysfunction relative to E4(-) subjects (for review see [16]) and therefore may not metabolize ketone bodies as well. Alternatively, differential insulin signaling seen in AD patients based on E4 carriage status may affect transport and metabolism of ketone bodies.

To gain further insight into this phenomenon, and to provide direction for future research, other genetic markers were tested for their ability to modulate performance on the ADAS-Cog test during induced ketosis in mild to moderate AD patients. Here we report the effects of polymorphisms in insulin degrading enzyme (*IDE*) and interleukin 1-beta (*IL1B*) on ADAS-Cog scores after 45 and 90 days of a ketogenic therapy followed by a two week washout, day 104. A report detailing the present study population, the overall results, and the *APOE4 *effects on cognitive outcomes was previously published [15].

Ketosis was induced by the administration of AC-1202, a formulation of medium chain triglycerides (MCTs). MCTs are triglycerides with fatty acid chains of between 5 and 12 carbons The catabolism of MCTs differs substantially from the more common long chain triglycerides (LCTs). MCTs are immune to the regulation of LCT catabolism and are well known for their ability to induce ketosis after oral administration (for review see [17]).

Insulin degrading enzyme is a zinc binding metalloprotease encoded by the *IDE *gene located on chromosome 10. The Ide protein degrades a variety of short polypeptides including insulin and amyloid beta [18]. Polymorphisms in the region of the *IDE *gene have been implicated as risk factors in AD (for review see [2]). Neuroinflammation has also been considered a feature of AD and may influence risk and rate of progression of the disease [19]. Interleukin 1beta is a proinflammatory cytokine encoded by the *IL1B *gene located on chromosome 2. Interleukin levels are normally low in the CNS but are elevated after acute injury and in chronic neurodegenerative diseases such as AD [20]. In addition, for each marker, the interaction with E4 carriage status was examined.

## Methods

The analysis presented here is an extension of previously reported results of a study examining the induction of mild ketosis in patients with mild to moderate AD. As reported in the earlier study [15], an oral ketogenic compound, AC-1202, was tested in patients with probable mild to moderate AD to examine if ketosis could improve cognitive performance. AC-1202 was administered daily for 90 days in 152 subjects in a US-based, randomized, double-blind, placebo-controlled, parallel-group study. Subjects were not asked to change their diets and continued taking approved AD medications. The results of the 90-day study found a significant difference between AC-1202 and Placebo in mean change from Baseline in ADAS-Cog score on Day 45. Based on previous acute dosing study [14], results of cognitive tests were stratified by *APOE4 *carriage status. Significant differences were reported between AC-1202 and Placebo in mean change from Baseline in ADAS-Cog score on both Day 45 and Day 90 among participants who were non-carriers of the *APOE4 *allele. Supporting the improvement relative to placebo by administration of AC-1202, a significant pharmacologic response was observed between serum β-hydroxybutyrate levels and change in ADAS-Cog scores in the non-carriers of *APOE4*. Detailed description of this study and its outcomes has been previously published [15].

### Ethics

The trial was carried-out in accordance with the principles of the Declaration of Helsinki and with institutional review board approval (Essex Institutional Review Board, Lebanon, NJ). Subjects and their caregivers provided written informed consent, which included an optional written provision for genotyping. For genetic consent, participants could consent to be tested for *APOE *genotype only, for any additional DNA markers only, for both, or for neither. Genetic consent was not required for entry into the study. Genetic information was not shared with physicians, site personnel, or study participants.

### Registration

This trial was registered with ClinicalTrials.gov, registry number NCT00142805, information available at http://clinicaltrials.gov/ct2/show/NCT00142805.

### Study design

This was a randomized, double-blind, placebo-controlled, parallel, multi-center trial sponsored by Accera, Inc. of Broomfield, CO. It was conducted between October 5, 2004 and June 29, 2006 at 23 treatment centers located within the United States. The study recruited outpatients with a diagnosis of probable AD of mild to moderate severity according to National Institute of Neurological and Communicative Disorders and Stroke and the Alzheimer's Disease and Related Disorders Association (NINCDS-ADRDA) and DSM IV criteria, with a MMSE score of between 14 and 24 (inclusive) at Screen. A CT or MRI within 24 months prior to Screen had to show no signs of tumor, structural abnormality, or degenerative disease. Subjects were required to have a Modified Hachinski Ischemia Scale score ≤4. Participants were randomized to receive either daily doses of AC-1202 or matching Placebo for 90 days. To acclimate participants to investigational product, subjects received a one-half dose daily during the first week of the study. After this one week titration, participants were instructed to take a full dose. A full dose of active contained 20 grams of MCTs. The effects of the intervention on cognitive performance were measured at 45 and 90 days post-Baseline and after a two week washout on Day 104. Primary cognitive outcome was change from Baseline in ADAS-Cog scores compared to Placebo. Participants in the study were allowed to remain on currently prescribed AD medications, provided they were on stable dosing for at least three months prior to enrollment and did not change dosing during the course of the study. Most participants (> 75%) were on one or more currently approved AD medications. Placebo and AC-1202 groups were well matched for demographic parameters. For detailed description of study and participants see Henderson et. al. [15].

### Subject disposition

**Figure 1:**
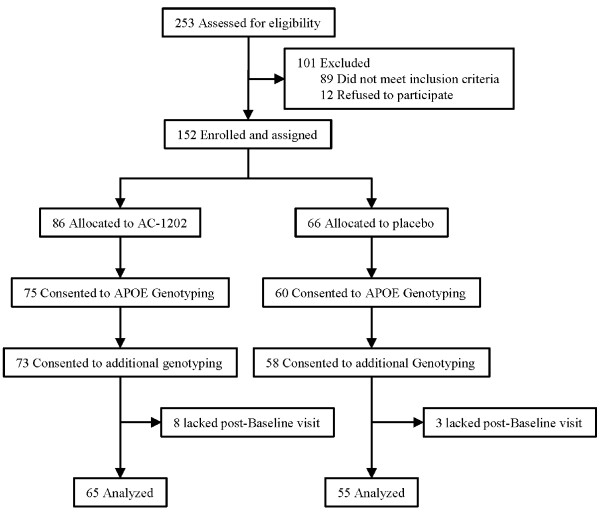
**Subject Disposition**.

Two hundred fifty-three subjects were screened at 23 clinical sites located within the United States. One hundred one of these participants did not meet inclusion/exclusion criteria or refused to participate in the study. One hundred fifty-two participants were enrolled in the study. At their discretion, participants could consent to *APOE *genotyping only, additional genetic markers only, or both. One hundred thirty-five of the enrolled subjects consented to genotyping for the *APOE *locus. One hundred thirty-one subjects consented to both *APOE *genotyping and additional markers. Of these 131 subjects, 11 lacked a post-Baseline visit and no ADAS-Cog data was available for analysis (Figure 1).

### Study material

AC-1202 (caprylic triglyceride, NeoBee 895, Stepan Chemical Company) is a structured medium chain triglyceride (MCT) in which greater than 95% of the fatty acids are caprylic acid (C8:0). NeoBee 895 is an approved food additive. Consumption of MCTs may give rise to gastrointestinal distress such as cramping, nausea and diarrhea. To improve tolerability, caprylic triglyceride was formulated as an emulsified spray dried powder consisting of 33% AC-1202 (NeoBee 895), 64% gum Acacia (Instagum, CNI) and 2.6% syloid (244FP, Grace Davison). Placebo was formulated to be isocaloric to the active formulation and consisted of a mixture of 51% gum acacia, 37% dextrose, 10% safflower oil and 2% syloid (prepared by The Chemins Company). Investigational product was given as a powder packaged in 30 gram sachets containing either active (equivalent to 10 grams of AC-1202) or matching Placebo. A full dose consisted of two sachets (equivalent to 20 grams of AC-1202). Product was reconstituted in water or other liquids.

### β-hydroxybutyrate testing

Blood samples were taken for serum BHB levels prior to dosing and 2 hr post-dosing. Levels of serum β-hydroxybutyrate were determined using the Stanbio Laboratory β-hydroxybutyrate test kit (StanBio Inc). Briefly, in the presence of NAD, ß-hydroxybutyrate is converted to acetoacetate and NADH by the enzyme ß-hydroxybutyrate dehydrogenase. The NADH produced reacts with p-iodonitrotetrazolium in the presence of diaphorase to produce a color that is read at 505 nm to determine the concentration.

### Genotyping

High molecular weight DNA was isolated from whole blood using standard techniques. Apolipoprotein E (*APOE*) epsilon 2, 3 and 4 genotyping was performed using allele specific extension as previously described [21]. For single nucleotide polymorphisms, genotyping was performed by amplification of genomic DNA isolated from whole blood and sequencing samples using standard procedures. Primer pair sequences used for amplification and sequencing were: *IDE *rs2251101 CAGCACTTTAGGAGGCCAAG/CTGCCCTTACAGGGATGAAA; *IL1B *rs1143627 and rs16944 CACAAAGAGGCAGAGAGACAGA/GTCTTGCAGGGTTGTGTGAG. In some cases an unambiguous genotype could not be determined and no genotype was assigned.

### Cognitive Testing

Subjects were administered the ADAS-Cog test at Baseline and Days 45, 90 and 104. The ADAS-Cog is one of the most widely used scales for anti-dementia drugs in the United States. The ADAS-Cog subscale consists of 11 tasks measuring cognitive abilities in memory, language, orientation, and praxis, with a total score ranging from 0 (no impairment) to 70 (severe impairment). The higher the ADAS-Cog score, the more impaired the subject. Therefore, lowering of the ADAS-Cog score is a measure of cognitive improvement.

### Statistical Methods

Analysis was done comparing change from Baseline in ADAS-Cog scores using reported scores between Active and Placebo treated groups on Days 45, 90 and 104. Only actual scores for each time point were used in this analysis. No data was imputed. An overall two way analysis of variance was used to evaluate cognitive scores by genotype and treatment interactions at Days 45, 90 and 104.

## Results and Discussion

### Single Nucleotide Polymorphisms (SNPs) effects on ADAS-Cog

Single nucleotide polymorphisms (SNPs) rs1143627 and rs16944 in the promoter region of *IL1B *and rs2251101 in the 3' untranslated region of *IDE *were analyzed for their effects on ADAS-Cog scores after administration of AC-1202 or Placebo. The genotypic frequencies of the SNPs in the study population are listed in Table 1. Cognitive outcomes associated with common *APOE *genotypes were also examined. Mean change from Baseline in ADAS-Cog scores are shown in Table 2.

**Table 1:** Distribution of genotypes

gene	SNP	AC-1202			Placebo	
		**Allele**	**Count**	**Frequency**	**Count**	**Frequency**

*IL1B*	rs1143627	C	6	0.098	9	0.173
		Het	27	0.443	17	0.327
		T	28	0.459	26	0.500
		Total	61	1	52	1
*IL1B*	rs16944	C	28	0.459	26	0.500
		Het	27	0.443	17	0.327
		T	6	0.098	9	0.173
		Total	61	1	52	1
*IDE *	rs2251101	C	9	0.138	7	0.127
		Het	21	0.323	25	0.454
		T	35	0.538	23	0.418
		Total	65	1	55	1

**Table 2:** Change in ADAS-Cog scores

Day 45 Change from Baseline								
**Gene**	**SNP/Allele**	**Genotype**	**AC1202**	**n**	**Placebo**	**n**	p-value*	**Difference (CI)**

*APOE*	*APOE*	3/2	-1.222	3	5.167	2	0.182	6.389(-3.050, 15.828)
*APOE*	*APOE*	3/3	-2.317	20	2.999	23	**0.001**	5.315(2.154, 8.476)
*APOE*	*APOE*	4/2	2.778	3	.	0	na	na
*APOE*	*APOE*	4/3	1.107	28	2.175	19	0.492	1.068(-2.005, 4.142)
*APOE*	*APOE*	4/4	-1.667	3	-1.167	10	0.884	0.500(-6.306, 7.306)
*IDE *	rs2251101	C	2.375	8	-0.905	7	0.138	3.280(-1.076, 7.635)
*IDE *	rs2251101	Het	-1.647	17	2.317	23	**0.004**	3.964(1.273, 6.656)
*IDE *	rs2251101	T	-0.711	30	1.290	23	0.092	2.001(-0.331, 4.333)
*IDE *c/c	*IDE *c/c (-)	*IDE *-	-1.050	47	1.804	46	**0.002**	2.853(1.100, 4.606)
*IDE *c/c	*IDE *c/c (+)	*IDE *+	2.375	8	-0.905	7	0.140	3.280(-1.094, 7.654)
*IL1B*	rs1143627	C	1.000	5	2.481	9	0.545	1.481(-3.365, 6.328)
*IL1B*	rs1143627	Het	-1.097	24	-0.020	17	0.439	1.078(-1.677, 3.832)
*IL1B*	rs1143627	T	-0.768	23	2.026	24	**0.031**	2.795(0.259, 5.330)
*IL1B*	rs16944	C	-0.768	23	2.026	24	**0.031**	2.795(0.259, 5.330)
*IL1B*	rs16944	Het	-1.097	24	-0.020	17	0.439	1.078(-1.677, 3.832)
*IL1B*	rs16944	T	1.000	5	2.481	9	0.545	1.481(-3.365, 6.328)

Day 90 Change from Baseline								

*APOE*	*APOE*	3/2	3.667	1	6.333	1	0.737	2.667(-13.098, 18.431)
*APOE*	*APOE*	3/3	-2.579	19	1.398	20	**0.029**	3.977(0.406, 7.548)
*APOE*	*APOE*	4/2	-3.667	2	.	0	na	na
*APOE*	*APOE*	4/3	1.302	21	0.471	17	0.651	0.831(-2.806, 4.468,)
*APOE*	*APOE*	4/4	1.667	2	0.867	10	0.854	0.800(-7.835, 9.435)
*IDE *	rs2251101	C	3.905	7	-1.857	7	**0.047**	5.762(0.074, 11.450)
*IDE *	rs2251101	Het	-2.769	13	2.944	19	**0.004**	5.713(1.883, 9.543)
*IDE *	rs2251101	T	-0.931	24	0.446	21	0.392	1.377(-1.803, 4.556)
*IDE *c/c	*IDE *c/c (-)	*IDE *-	-1.577	37	1.633	40	**0.011**	3.209(0.766, 5.652)
*IDE *c/c	*IDE *c/c (+)	*IDE *+	3.905	7	-1.857	7	**0.049**	5.762(0.038, 11.486)
*IL1B*	rs1143627	C	-1.083	4	1.815	9	0.396	2.898(-3.859, 9.656)
*IL1B*	rs1143627	Het	-0.193	19	-0.044	15	0.940	0.149(-3.735, 4.033)
*IL1B*	rs1143627	T	-1.947	19	1.748	20	**0.044**	3.696(0.093, 7.298)
*IL1B*	rs16944	C	-1.947	19	1.748	20	**0.044**	3.696(0.093, 7.298)
*IL1B*	rs16944	Het	-0.193	19	-0.044	15	0.940	0.149(-3.735, 4.033)
*IL1B*	rs16944	T	-1.083	4	1.815	9	0.396	2.898(-3.859, 9.656)

Day 104 Change from Baseline								

*APOE*	*APOE*	3/2	-1.333	3	2.333	2	0.474	3.667(-6.443, 13.776)
*APOE*	*APOE*	3/3	-0.812	23	1.384	21	0.196	2.196(-1.147, 5.538)
*APOE*	*APOE*	4/2	0.889	3	.	0	na	na
*APOE*	*APOE*	4/3	0.821	26	-0.722	18	0.370	1.543(-1.853, 4.938)
*APOE*	*APOE*	4/4	-1.083	4	0.933	10	0.543	2.017(-4.535, 8.568)
*IDE *	rs2251101	C	3.762	7	0.952	7	0.331	2.810(-2.895, 8.514)
*IDE *	rs2251101	Het	-2.169	18	0.317	20	0.158	2.485(-0.982, 5.953)
*IDE *	rs2251101	T	-0.029	33	0.867	22	0.547	0.896(-2.042, 3.834)
*IDE *c/c	*IDE *c/c (-)	*IDE *-	-0.784	51	0.605	42	0.217	1.389(-0.828, 3.606)
*IDE *c/c	*IDE *c/c (+)	*IDE *+	3.762	7	0.952	7	0.330	2.810(-2.878, 8.497)
*IL1B*	rs1143627	C	0.778	6	0.407	9	0.898	0.370(-5.373, 6.114)
*IL1B*	rs1143627	Het	-0.376	24	0.524	14	0.627	0.900(-2.765, 4.565)
*IL1B*	rs1143627	T	-0.720	25	0.380	23	0.490	1.100(-2.049, 4.248)
*IL1B*	rs16944	C	-0.720	25	0.380	23	0.490	1.100(-2.049, 4.248)
*IL1B*	rs16944	Het	-0.376	24	0.524	14	0.627	0.900(-2.765, 4.565)
*IL1B*	rs16944	T	0.778	6	0.407	9	0.898	0.370(-5.373, 6.114)

*p-value < 0.05 bold, < 0.01 bold and underlined. na = not available

For SNP rs2251101 near the *IDE *gene, heterozygous C/T subjects administered AC-1202 demonstrated significant improvement relative to Placebo at Days 45 (Δ3.96; CI 1.27- 6.66; p = 0.004) and Day 90 (Δ5.71; CI 1.88-9.54; p = 0.004). When participants were categorized as either *IDE *rs2251101 C/C(-) or *IDE *rs2251101 C/C(+) based on their carriage status of the C/C genotype, significant improvement was found in *IDE *rs2251101 C/C(-) subjects administered AC-1202 relative to Placebo at Days 45 (Δ2.85; CI 1.10-4.61; p = 0.002) and Day 90 (Δ3.21;CI 0.766-5.652; p = 0.011) (Table 2).

For SNPs rs1143627 and rs16944 in the promoter region of *IL1B *gene, identical significant effects were seen for rs1143627 T/T and rs16944 C/C carriers. Both genotypes demonstrated significant improvement among those administered AC-1202 relative to Placebo at Days 45 (Δ2.79; CI 0.26-5.33; p = 0.031) and Day 90 (Δ3.70; CI 0.09-7.30; p = 0.044). Note, rs1143627 T and rs16944 C were in complete linkage disequilibrium in the sample population.

No significant genotype interactions for any SNPs were found between AC-1202 and Placebo at Day 104 (Table 2).

### *APOE *genotype effects on ADAS-Cog

Among *APOE *genotypes, homozygous carriers of the epsilon 3 allele administered AC-1202 demonstrated significant improvement relative to Placebo at Days 45 (Δ5.31; CI 2.15- 8.48; p = 0.0012) and Day 90 (Δ3.98; CI 0.41-7.55; p = 0.030). Carriers of the E4 allele did not demonstrate significant effects on ADAS-Cog scores. Subjects who carried a single E4 allele, for example E4/E3 genotype did not differ significantly from Placebo at either Day 45 or Day 90 (See Table 2). Consistent with earlier results, when participants were categorized as either E4(+) or E4(-) based on their carriage status of the epsilon 4 allele, significant improvement were found in E4(-) subjects administered AC-1202 relative to Placebo at Days 45 (Δ5.35; CI 2.37-8.321; p = 0.0006) and Day 90 (Δ3.9; CI 0.45-7.35; p = 0.027). Due to the small number of E4/E4 carriers, it was not possible to examine dosage effects of E4 on ADAS-Cog scores.

Note, the dataset used in the present study included only ADAS-Cog scores from a subset of participants who signed genetic consent for additional markers and hence the means and p-values differ slightly from the population who consented to *APOE *testing as previously reported [15]. However, the overall findings of significance did not change.

### Interactions with *APOE4*

Previous studies have demonstrated a pharmacogenetic response in cognitive performance by induction of ketosis based on *APOE4 *carriage status. In the present study, each of the SNPs were examined for an interaction between the SNP genotype and E4 carriage status, as defined as either E4(-) or E4(+).

Evidence of possible interactions with *APOE4 *were defined as genetic combinations that produced enhanced cognitive improvement at Day 45, Day 90 and Day 104 when compared to the cognitive results for E4(-) subjects alone. As described above, among E4(-) subjects administered AC-1202, change from Baseline scores on ADAS-Cog improved an average of -2.16 points on Day 45, -2.27 points on Day 90, and -0.87 points on Day 104.

**Figure 2:**
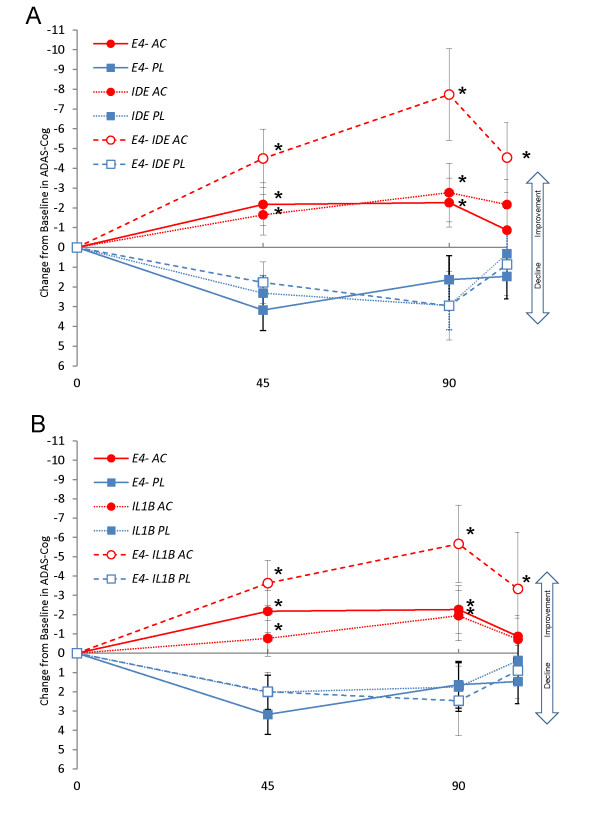
**Change from Baseline in ADAS-Cog scores among responder genotypes over time**. Red markers represent mean change from Baseline among subjects administered AC-1202. Blue markers represent mean change from Baseline among subjects administered Placebo. Error bars represent standard error of the mean. A Solid lines and solid markers represent subjects who are *APOE4*(-). Dotted lines and solid markers represent mean scores of subjects who are heterozygous for the *IDE *SNP rs2251101. Dashed lines and open markers represent mean scores of subjects who were E4(-) and heterozygous for the *IDE *SNP rs2251101. B Solid lines and solid markers represent subjects who are *APOE4*(-). Dotted lines and solid markers represent mean scores of subjects who are homozygous for the *IL1B *SNP rs1143627 T allele. Dashed lines and open markers represent mean scores of subjects who were E4(-) and homozygous for the *IL1B *SNP rs1143627 T allele. Asterisks represent significant difference between AC-1202 and Placebo means (p-value < 0.05).

Specific genotype combinations of *IDE *and E4(-) as well as *IL1B *and E4(-) produced additive improvements in cognitive performance. Among E4(-) subjects administered AC-1202 who were also heterozygous C/T for the *IDE *rs2251101SNP, scores on ADAS-Cog improved from Baseline an average of -4.5 points on Day 45, -7.73 points on Day 90, and -4.5 points on Day 104, all of which were significantly different from Placebo means (p < 0.05) (Figure 2A, Table 3). Among E4(-) subjects administered AC-1202 who were also homozygous for T/T for the *IL1B *rs1143627 SNP (all of these subjects were also homozygous for the C/C allele of rs16944), scores on ADAS-Cog improved from Baseline an average of -3.6 points on Day 45, -5.7 points on Day 90 and -3.3 points on Day 104, all of which were significantly different from Placebo means (p < 0.05) (Figure 2B, Table 3). No significant differences between Active and Placebo were found among E4(+) subjects and any genotypes of *IDE *or *IL1B *(Table 4).

**Table 3:** Change in ADAS-Cog scores among *APOE4*(-) participants

Day 45 Change from Baseline								
**gene**	**SNP**	**Allele**	**AC1202**	**n**	**Placebo**	**n**	p-value*	**Difference(CI)**

*IDE *	*IDE*-	*IDE *c/c (-)	-2.772	19	2.071	23	**0.000**	4.843(2.568, 7.118)
*IDE *	*IDE*+	*IDE *c/c (+)	0.667	4	-0.667	1	0.745	1.333(-6.870, 9.536)
*IDE *	rs2251101	C	0.667	4	-0.667	1	0.744	1.333(-6.867, 9.534)
*IDE *	rs2251101	Het	-4.500	6	1.775	12	**0.001**	6.275(2.608, 9.942)
*IDE *	rs2251101	T	-1.974	13	2.394	11	**0.005**	4.368(1.364, 7.373)
*IL1B*	rs1143627	C	0.333	3	4.333	3	0.192	4.000(-2.103, 10.103)
*IL1B*	rs1143627	Het	-2.083	8	0.524	7	0.180	2.607(-1.261, 6.476)
*IL1B*	rs1143627	T	-3.633	10	1.997	13	**0.001**	5.631(2.487, 8.775)
*IL1B*	rs16944	C	-3.633	10	1.997	13	**0.001**	5.631(2.487, 8.775)
*IL1B*	rs16944	Het	-2.083	8	0.524	7	0.180	2.607(-1.261, 6.476)
*IL1B*	rs16944	T	0.333	3	4.333	3	0.192	4.000(-2.103, 10.103)

Day 90 Change from Baseline								

*IDE *	*IDE*-	*IDE *c/c (-)	-3.729	16	2.156	19	**0.003**	5.885(2.192, 9.578)
*IDE *	*IDE*+	*IDE *c/c (+)	3.583	4	-5.333	1	0.146	8.917(-3.252, 21.086)
*IDE *	rs2251101	C	3.583	4	-5.333	1	0.132	8.917(-2.834, 20.667)
*IDE *	rs2251101	Het	-7.733	5	2.956	9	**0.001**	10.689(4.827, 16.551)
*IDE *	rs2251101	T	-1.909	11	1.437	10	0.148	3.346(-1.246, 7.938)
*IL1B*	rs1143627	C	-1.000	3	3.222	3	0.370	4.222(-5.241, 13.685)
*IL1B*	rs1143627	Het	-0.250	8	-0.389	6	0.964	0.139(-6.120, 6.398)
*IL1B*	rs1143627	T	-5.667	8	2.463	10	**0.005**	8.130(2.633, 13.627)
*IL1B*	rs16944	C	-5.667	8	2.463	10	**0.005**	8.130(2.633, 13.627)
*IL1B*	rs16944	Het	-0.250	8	-0.389	6	0.964	0.139(-6.120, 6.398)
*IL1B*	rs16944	T	-1.000	3	3.222	3	0.370	4.222(-5.241, 13.685)

Day 104 Change from Baseline								

*IDE *	*IDE*-	*IDE *c/c (-)	-1.909	22	1.686	21	**0.026**	3.595(0.451, 6.739)
*IDE *	*IDE*+	*IDE *c/c (+)	4.833	4	-3.333	1	0.160	8.167(-3.356, 19.690)
*IDE *	rs2251101	C	4.833	4	-3.333	1	0.152	8.167(-3.117, 19.451)
*IDE *	rs2251101	Het	-4.546	8	0.867	10	**0.028**	5.413(0.625, 10.200)
*IDE *	rs2251101	T	-0.402	14	2.430	11	0.167	2.833(-1.234, 6.899)
*IL1B*	rs1143627	C	1.444	3	-0.333	3	0.670	1.778(-6.598, 10.153)
*IL1B*	rs1143627	Het	0.478	9	1.611	6	0.674	1.133(-4.273, 6.540)
*IL1B*	rs1143627	T	-3.333	12	0.894	12	**0.048**	4.228(0.040, 8.415)
*IL1B*	rs16944	C	-3.333	12	0.894	12	**0.048**	4.228(0.040, 8.415)
*IL1B*	rs16944	Het	0.478	9	1.611	6	0.674	1.133(-4.273, 6.540)
*IL1B*	rs16944	T	1.444	3	-0.333	3	0.670	1.778(-6.598, 10.153)

*p-value < 0.05 bold, < 0.01 bold and underlined.

**Table 4:** Change in ADAS-Cog scores among *APOE4*(+) participants

Day 45 Change from Baseline								
**gene**	**SNP**	**Allele**	**AC1202**	**n**	**Placebo**	**n**	**p-value**	**Difference(CI)**

*IDE*	*IDE*-	*IDE *c/c (-)	0.119	28	1.536	23	0.276	1.417(-1.163, 3.998)
*IDE*	*IDE*+	*IDE *c/c (+)	4.083	4	-0.944	6	0.094	5.028(-0.892, 10.947)
*IDE*	rs2251101	C	4.083	4	-0.944	6	0.091	5.028(-0.827, 10.883)
*IDE*	rs2251101	Het	-0.091	11	2.909	11	0.126	3.000(-0.868, 6.868)
*IDE*	rs2251101	T	0.255	17	0.278	12	0.989	0.023(-3.397, 3.443)
*IL1B*	rs1143627	C	2.000	2	1.556	6	0.908	0.444(-7.234, 8.123)
*IL1B*	rs1143627	Het	-0.604	16	-0.400	10	0.914	0.204(-3.587, 3.995)
*IL1B*	rs1143627	T	1.436	13	2.061	11	0.746	0.625(-3.228, 4.477)
*IL1B*	rs16944	C	1.436	13	2.061	11	0.746	0.625(-3.228, 4.477)
*IL1B*	rs16944	Het	-0.604	16	-0.400	10	0.914	0.204(-3.587, 3.995)
*IL1B*	rs16944	T	2.000	2	1.556	6	0.908	0.444(-7.234, 8.123)

Day 90 Change from Baseline								

*IDE *	*IDE*-	*IDE *c/c (-)	0.063	21	1.159	21	0.508	1.095(-2.211, 4.402)
*IDE *	*IDE*+	*IDE *c/c (+)	4.333	3	-1.278	6	0.143	5.611(-1.965, 13.187)
*IDE *	rs2251101	C	4.333	3	-1.278	6	0.143	5.611(-1.961, 13.183)
*IDE *	rs2251101	Het	0.333	8	2.933	10	0.308	2.600(-2.479, 7.679)
*IDE *	rs2251101	T	-0.103	13	-0.455	11	0.872	0.352(-4.035, 4.739)
*IL1B*	rs1143627	C	-1.333	1	1.111	6	0.687	2.444(-9.715, 14.603)
*IL1B*	rs1143627	Het	-0.152	11	0.185	9	0.894	0.337(-4.723, 5.396)
*IL1B*	rs1143627	T	0.758	11	1.033	10	0.910	0.276(-4.643, 5.194)
*IL1B*	rs16944	C	0.758	11	1.033	10	0.910	0.276(-4.643, 5.194)
*IL1B*	rs16944	Het	-0.152	11	0.185	9	0.894	0.337(-4.723, 5.396)
*IL1B*	rs16944	T	-1.333	1	1.111	6	0.687	2.444(-9.715, 14.603)

Day 104 Change from Baseline								

*IDE *	*IDE*-	*IDE *c/c (-)	0.069	29	-0.476	21	0.733	0.545(-2.646, 3.737)
*IDE *	*IDE*+	*IDE *c/c (+)	2.333	3	1.667	6	0.866	0.667(-7.210, 8.543)
*IDE *	rs2251101	C	2.333	3	1.667	6	0.868	0.667(-7.357, 8.690)
*IDE *	rs2251101	Het	-0.267	10	-0.233	10	0.990	0.033(-5.041, 5.108)
*IDE *	rs2251101	T	0.246	19	-0.697	11	0.662	0.943(-3.356, 5.242)
*IL1B*	rs1143627	C	0.111	3	0.778	6	0.871	0.667(-7.534, 8.868)
*IL1B*	rs1143627	Het	-0.889	15	-0.292	8	0.814	0.597(-4.480, 5.675)
*IL1B*	rs1143627	T	1.692	13	-0.182	11	0.432	1.874(-2.877, 6.625)
*IL1B*	rs16944	C	1.692	13	-0.182	11	0.432	1.874(-2.877, 6.625)
*IL1B*	rs16944	Het	-0.889	15	-0.292	8	0.814	0.597(-4.480, 5.675)
*IL1B*	rs16944	T	0.111	3	0.778	6	0.871	0.667(-7.534, 8.868)

### Changes in serum β-hydroxybutyrate levels

To examine if differences in cognitive performance were due to differences in circulating ketone body levels, serum β-hydroxybutyrate (BHB) levels were compared between potential responder genotypes. Serum BHB levels among subjects receiving AC-1202 were compared between genotypes at Baseline, Day 45 and Day 90. At Baseline 1/2 dose (10 grams of AC-1202) was administered and on Days 45 and 90 full dose (20 grams of AC-1202). Significant differences were found at the Baseline visit between E4 carriers and non-carriers (p-value 0.03). However, this finding did not reproduce at later time points. Among E4(-) and *IDE *and *IL1B *genotypes administered AC-1202, there were no significant differences between BHB levels at any study visit (Table 5).

**Table 5:** β-hydroxybutyrate levels among responder genotypes

*APOE4 *status	SNP	Genotype	N	Mean BHB mM	Std Error	Lower 95%	Upper 95%	p-value*
Baseline Post-dose (1/2 dose)								

E4-			29	0.121	0.014	0.093	0.148	
E4+			38	0.161	0.012	0.137	0.185	0.030
**E4-**	***IL1B *rs1143627**	**C/C**	**3**	**0.103**	**0.032**	**0.037**	**0.170**	
**E4-**	***IL1B *rs1143627**	**C/T**	**9**	**0.126**	**0.019**	**0.087**	**0.164**	
**E4-**	***IL1B *rs1143627**	**T/T**	**14**	**0.120**	**0.015**	**0.089**	**0.151**	**0.838**
E4-	*IDE *rs2251101	C/C	5	0.096	0.025	0.045	0.147	
E4-	*IDE *rs2251101	C/T	9	0.134	0.018	0.097	0.172	
E4-	*IDE *rs2251101	T/T	15	0.121	0.014	0.091	0.150	0.471

Day 45 Post-dose (full dose)								

E4-			23	0.334	0.047	0.240	0.428	
E4+			33	0.355	0.039	0.276	0.434	0.730
**E4-**	***IL1B *rs1143627**	**C/C**	**3**	**0.227**	**0.102**	**0.013**	**0.441**	
**E4-**	***IL1B *rs1143627**	**C/T**	**8**	**0.431**	**0.062**	**0.300**	**0.562**	
**E4-**	***IL1B *rs1143627**	**T/T**	**10**	**0.278**	**0.056**	**0.161**	**0.395**	**0.132**
E4-	*IDE *rs2251101	C/C	4	0.470	0.099	0.263	0.677	
E4-	*IDE *rs2251101	C/T	6	0.315	0.081	0.146	0.484	
E4-	*IDE *rs2251101	T/T	13	0.301	0.055	0.186	0.416	0.338

Day 90 Post-dose(full dose)								

E4-			20	0.301	0.076	0.147	0.454	
E4+			22	0.471	0.072	0.325	0.617	0.111
**E4-**	***IL1B *rs1143627**	**C/C**	**3**	**0.193**	**0.144**	**-0.111**	**0.498**	
**E4-**	***IL1B *rs1143627**	**C/T**	**8**	**0.378**	**0.088**	**0.191**	**0.564**	
**E4-**	***IL1B *rs1143627**	**T/T**	**8**	**0.296**	**0.088**	**0.110**	**0.483**	**0.544**
E4-	*IDE *rs2251101	C/C	4	0.123	0.113	-0.116	0.361	
E4-	*IDE *rs2251101	C/T	5	0.470	0.101	0.257	0.683	
E4-	*IDE *rs2251101	T/T	11	0.288	0.068	0.144	0.432	0.099

*ANOVA of means

Induction of ketosis by the oral administration of the ketogenic compounds MCTs improved cognitive performance in mild to moderate AD patients relative to Placebo. Yet, the improvement was largely restricted to non-carriers of the AD risk factor *APOE4 *[14,15]. In the present study, additional genetic markers were examined for their ability to influence cognitive performance at specific time points during a 90 day dosing of MCTs. In general, the low number of subjects in the study did not allow for definitive analysis, and the results should be considered exploratory.

Ketone bodies are produced mainly by the liver from fatty acids (FA) during periods of low carbohydrate availability. Ketogenesis and the utilization of ketone bodies is regulated at several key steps that, under normal feeding conditions, prevent substantial amounts of ketone bodies from being produced. Ketogenesis requires abundant circulating free fatty acid (FFA) levels for oxidation in the liver. Thus, conditions, such as high carbohydrate diet and elevated insulin signaling prevent ketone body production.

Ketone bodies have several properties that make them attractive for treating neurodegenerative disorders. During starvation conditions ketone bodies can substitute for the majority of the brain's energy requirements [11]. Studies with infused ketone bodies have demonstrated that even in the presence of normal glucose, the brain will metabolize ketone bodies [22]. Therefore, in conditions where glucose use is impaired, ketone bodies may offer a substitute fuel. This is the rationale for the successful use of ketogenic diets in GLUT1 deficiency syndrome, where the brain cannot transport sufficient glucose for normal function and ketones can substitute for the lack of glucose [23]. Similarly, the regional decreases in CMR(glc) seen in AD may benefit from the exogenous supplementation with ketone bodies (for review see [12]).

The SNP rs2251101 is located in the 3' un-translated region of the *IDE *gene. The C allele has been associated with a putative reduction of function haplotype of IDE. Homozygous carriers of the C allele were found to present high fasting and post-prandial insulin levels, as well as greater body mass index, suggestive of low Ide activity [24]. In the present study, heterozygous C/T carriers as well as grouped non-C/C carriers administered AC-1202 had significantly improved ADAS-Cog scores at Days 45 and 90 relative to Placebo subjects of the same genotype. Homozygous C/C carriers may have reduced Ide activity and hence higher insulin levels. High insulin levels may reduce levels of monocarboxylate transporters and inhibit the ability to respond to induced ketosis. It is well recognized that insulin signaling promotes fatty acid storage and reduces fatty acid oxidation, while conditions of low insulin signaling, such as fasting, promote ketogenesis and ketolysis. This mechanism is likely to operate in the brain [25].

Two SNPs (rs1143627 and rs16944) found in the promoter region of the *IL1B *gene have been associated with clinically observed differences in the levels of Il1β protein in vivo. The SNP rs1143627 C/T is located at position -31 in the putative TATA box of the *IL1B *gene. The SNP rs16944 C/T is located at position -511. A haplotype, composed of the T allele at -511 and the C allele at -31, is significantly associated with a two to threefold increase in lipopolysaccharide (LPS) -induced Il1β protein secretion [26]. Thus, carriers of this haplotype may produce an enhanced inflammatory response and inhibit their ability to respond to ketosis, while non-carriers of this haplotype, (subjects who were homozygous for rs1143627 T and rs16944 C) showed significant response at Days 45 and 90.

**Figure 3:**
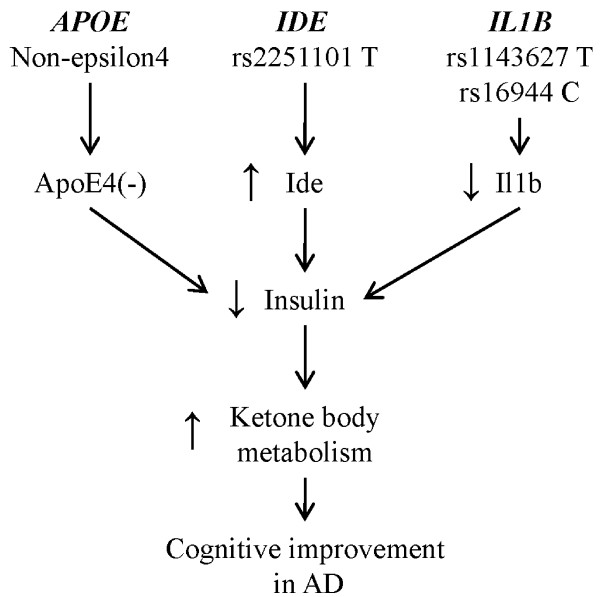
**A model of genotypic effects on ketone body metabolism in mild-to-moderate Alzheimer's disease**. Non-carriers of the *APOE4 *allele may have decreased insulin signaling allowing for increased ketone body metabolism. Carriers of the T allele of rs2251101 have elevated Ide activity relative to C carriers and therefore reduced insulin signaling. Carriers of the rs1143627 T and rs16944 C alleles have a reduced inflammatory response relative to rs1143627 C and rs16944 T carriers. This reduced inflammatory response may allow for improved ketone body metabolism. Each of the polymorphisms that reduce insulin signaling may allow for better response to induced ketosis in AD.

Similar to polymorphisms in IDE, enhanced inflammatory response may inhibit the ability of the cells to utilize ketone bodies by influencing insulin signaling. Inflammation is well recognized to diminish the rate of hepatic ketogenesis, possibly by increasing circulating insulin levels [27]. Notably, due to their unique metabolism, medium chain fatty acids, such as caprylic acid generated by AC-1202, are immune to the inflammatory inhibition of ketogenesis [28]. Of note, no significant differences in serum BHB levels were noted between *IL1B *rs1143627 and rs16944 genotypes. One mechanism by which elevated circulating insulin levels may inhibit the uptake of ketone bodies is by reduction of monocarboxylate transporter proteins, such as monocarboxyate transporter 1 (MCT1). Consistent with this view, inflammation of the intestine has been associated with decreased levels of MCT1 [29] and increased reliance on glucose [30], suggesting that inflammation could induce a shift away from ketone body metabolism towards glucose. In addition, inflammation may directly inhibit cells' ability to metabolize ketone bodies. For example, treating rats with LPS was found to cause nitration and reduced activity of the protein succinyl-CoA:3-oxoacid CoA transferase (SCOT; EC 2.8.3.5) [31]. SCOT catalyzes the formation of acetyl-CoA from acetoacetate and is the rate limiting step in the metabolism of ketone bodies. Therefore, inhibition of uptake or metabolism of ketone bodies by an enhanced inflammatory response due to polymorphisms in *IL1B *may reduce the ability of an AD patient to respond to induced ketosis (Figure 3).

Such a model may explain the additive effects seen among E4(-) subjects without the reduction of function *IDE *genotype rs2251101 C/C and without the proinflammatory genotype of *IL1B *rs1143627 C/C. Absence of these genotypes is predicted to lead to decreased insulin levels. The lower levels of insulin in an E4(-) background may be insufficient to overcome the mild insulin resistance and hence these subjects can respond to induced ketosis (Figure 3). In contrast, the relative insulin sensitivity of E4(+) subjects may prevent them from responding to ketosis in any genetic background [32,33].

When *IDE *and *IL1B *responder genotypes were examined in E4(+) carriers, no significant effects were observed, yet an additive or synergistic effect appears to be present in E4(-) subjects, suggesting that the effects seen in the overall genotyped population may have been driven by the strong response among E4(-) subjects who also carried the *IDE *or *IL1B *responder genotypes. This is notable by improvement in performance relative to the Placebo after the two week washout, which suggests that ketone bodies may confer a durable effect on cognition, possibly by improving mitochondrial efficiency and reducing oxidative damage [34] or through improvement in cerebral lipid environment [35].

## Conclusions

In conclusion, despite the relatively small size of this study, genetic influences on cognitive scores in response to induced ketosis were noted. The main modulator of induced ketosis appears to be the carriage status of *APOE4*. It may not be a coincidence that *APOE4 *is also the major genetic risk factor for late onset AD. The failure of *APOE4 *carriers to respond to ketosis may indicate a more insidious metabolic problem. *APOE4 *carriers may be overly reliant on glucose and hence, over a lifetime, cerebral neurons are deprived of the metabolic advantages conferred by ketone body metabolism and this may be crucial to etiology of AD [36]. Importantly, this type of pharmacogenomic profiling not only offers insights into the disease process, it also allows targeting of patients who are most likely to respond to therapy. In this way, better and more effective therapeutics can be developed.

## List of Abbreviations

ADAS-Cog: Alzheimer's Disease Assessment Scale-Cognitive subscale; AD: Alzheimer's disease; CMRglc: Cerebral Metabolic Rate of Glucose; MMSE: Mini Mental State Exam; NINCDS-ADRDA: National Institute of Neurological and Communicative Disorders and Stroke and the Alzheimer's Disease and Related Disorders Association; CT: computerized tomography; MRI: magnetic resonance imaging; MCT: medium chain triglyceride; CI: confidence interval; SCOT: succinyl-CoA:3-oxoacid CoA transferase.

## Statement of Competing interests

Some authors may benefit from this publication. Author SH is an employee of the sponsor of this trial, Accera Inc. SH has very minor stock ownership in Accera.

SH is the sole inventor of one issued patent (US 6835750) entitled: Use of medium chain triglycerides for the treatment and prevention of Alzheimer's disease and other diseases resulting from reduced neuronal metabolism II. SH and Accera have other published pending patent applications in this area: US 2002/0006959 A1, US 2003/0059824 A1, US 2006/0122270 A1, US 2008/0009467 A1, US 2006/0252775 A1, US 2007/0135376 A1, US 2008/0287372 A1, US 2007/0179197 A1.

## Authors' contributions

SH designed the research approach and wrote the paper. SH and JP analyzed data. All authors read and approved the final manuscript

## Pre-publication history

The pre-publication history for this paper can be accessed here:

http://www.biomedcentral.com/1471-2350/12/137/prepub
